# The antiandrogenic vinclozolin induces differentiation delay of germ cells and changes in energy metabolism in 3D cultures of fetal ovaries

**DOI:** 10.1038/s41598-020-75116-3

**Published:** 2020-10-22

**Authors:** Silvia González-Sanz, Odei Barreñada, Eduardo Rial, Miguel A. Brieño-Enriquez, Jesús del Mazo

**Affiliations:** 1 Department of Cellular and Molecular Biology, Centro de Investigaciones Biológicas Margarita Salas (CIB-CSIC), Ramiro de Maeztu, 9, 28040 Madrid, Spain; 2 Department of Structural and Chemical Biology, Centro de Investigaciones Biológicas Margarita Salas (CIB-CSIC), Ramiro de Maeztu, 9, 28040 Madrid, Spain; 3 Magee-Womens Research Institute, Department of Obstetrics, Gynecology and Reproductive Sciences, University of Pittsburgh School of Medicine, Pittsburgh, PA, USA

**Keywords:** Cell biology, Developmental biology, Molecular biology, Environmental sciences

## Abstract

Vinclozolin is a pesticide with antiandrogenic activity as an endocrine disruptor compound. Its effects upon the progression of primordial follicles were assessed in cultures of mouse fetal ovaries from the onset of meiotic differentiation of germ cells (13.5 days post coitum) and from both in vivo exposed mice and in vitro exposed ovaries. Exposure of ovaries to vinclozolin—at in vitro dosages ranging from 10 to 200 μM and in 3D ex vivo culture following in vivo exposure to 50 mg/kg bw/day—showed delays in meiocyte differentiation and in follicle growth, even at the lowest in vitro dose exposure. Immunofluorescent analysis showed the presence of the proteins MSY2 and NOBOX in the primary follicles but no difference in the level of protein signals or in the number of follicles in relation to treatment. However, assessing the cytological differentiation of germ cells by detecting the synaptonemal complex protein SYCP3, the exposure to vinclozolin delayed meiotic differentiation from both in vitro- and in vivo-exposed ovaries. These effects were concomitant with changes in the energy metabolism, detected as a relative increase of glycolytic metabolism in live-cell metabolic assays in exposed ovaries.

## Introduction

Mammalian oogenesis is a very complex process that requires perfect orchestration of intrinsic and extrinsic factors by germ cells in correlation with the surrounding granulosa and theca cells in the follicle^1,2^. The correct differentiation of primordial germ cells (PGCs) in meiosis and the progression to primordial follicles by interactions between germ and somatic cells are crucial to determine oocyte fate, oocyte quality, the ovarian reserve, and in consequence reproductive lifespan^3,4^.

In mouse ovaries, between 10.5 and 11.5 days post coitum (dpc), PGCs colonize the genital ridges. They then divide mitotically to increase the number of progenitor germ cells, initiating gonadal sex differentiation at about 12.5 dpc^5^. Mitotic division will continue within the ovary, where these divisions are characterized by incomplete cytokinesis, producing interconnected oogonia forming clusters of germ cells. Later, around 13.5 dpc, oogonia cells initiate meiosis and progress through meiotic prophase I up to dictyotene, remaining in that stage until meiotic resumption (after birth about 20 dpc). Meiotic prophase I is not fully synchronized, and during the first days of meiotic initiation, it is possible to observe mitotically active oogonia and oocytes having already entered meiotic prophase I during the first days of meiotic initiation^6^. The transition from cysts to individual follicles is initiated from 17.5 dpc, along with the beginning of follicular atresia, when only the surviving oocytes, as in other mammals, are surrounded by pre-granulosa cells^7–9^.

Various chemical compounds present in nature or of anthropogenic origin can alter gametogenesis. A well-known group of such chemicals are the endocrine-disrupting chemicals (EDCs), which have previously demonstrated health risk, specifically as reproductive toxicants, as reported in 2013 by the United Nations Environment Programme and the World Health Organization *(State of the Science of Endocrine Disrupting Chemicals – 2012.* www.who.int/ceh/publications/endocrine/en/index.html). The developmental dynamics of germ cell differentiation in mammalian females has hindered the analysis of some effects of the EDCs, particularly during the embryonic stages. Some critical studies have been carried out for specific EDCs in *C. elegans*^10,11^, mice^12^ and monkeys^13^. However, in mammals, the adverse effects of EDCs have been mainly assessed in males^14,15^. While the incidence of reproductive disorders in females caused by exposure to EDCs, especially during fetal life, is much higher than initially estimated, it has scarcely been assessed in either mice or humans^16,17^.

Ovarian function and folliculogenesis require a tight regulation and equilibrium of estrogenic and androgenic signals^18^. The effects of estrogens are well known but androgens have been more recently pointed out as crucial in ovarian function and folliculogenesis as well as one of the main causes of premature ovarian failure and polycystic ovarian syndrome^19^. Androgen production by granulosa and theca cells and their conversion to estrone and estradiol, mediated by BMP4-Smad signaling, have been recently identified in late folliculogenesis^20^. However, the effects of abnormal levels of androgens during early oogenesis are poorly understood. Vinclozolin (VZN) [3-(3,5-dichlorophenyl)-5-methyl-5-vinyl-1,3-oxazolidine-2,4-dione] is a fungicide widely used in agriculture and considered an EDC pesticide with antiandrogenic effects, both by itself and its metabolites^21^, blocking the androgen binding site of the receptors^22^. The adverse effects of VZN have been studied in spermatogenesis from a range of perspectives, including altered transgenerational effects on PGCs^23,24^. However, very limited studies have been performed on the effect of antiandrogen EDCs, particularly on VZN^25^, in oogenesis and early folliculogenesis.

Using in vivo and in vitro 3D cell culture systems^26^, we analyzed the disruptive effects of VZN on the development of the mouse ovary. We used ovaries from animals unexposed or exposed to vehicle (DMSO) (controls) and animals exposed to various concentrations of VZN. In particular, we evaluated the regulatory effects of VZN on the meiotic differentiation of PGCs, on folliculogenesis, and for the first time, on the mitochondrial energy metabolism of the female germ cell line, from PGCs to primary follicle. The results revealed both unexplored characteristics of normal development, such as in meiosis, and changes induced by the environmental pollutant VZN. Our study utilizing 3D cultures of fetal ovaries as a model, reveals that VZN affected the meiotic progression of PGCs and early folliculogenesis, leading to a delay in differentiation concomitant with an increase in the glycolytic pathway.

## Results

### Cytological and morphometric analysis of follicular progression in vitro

**Figure 1:**
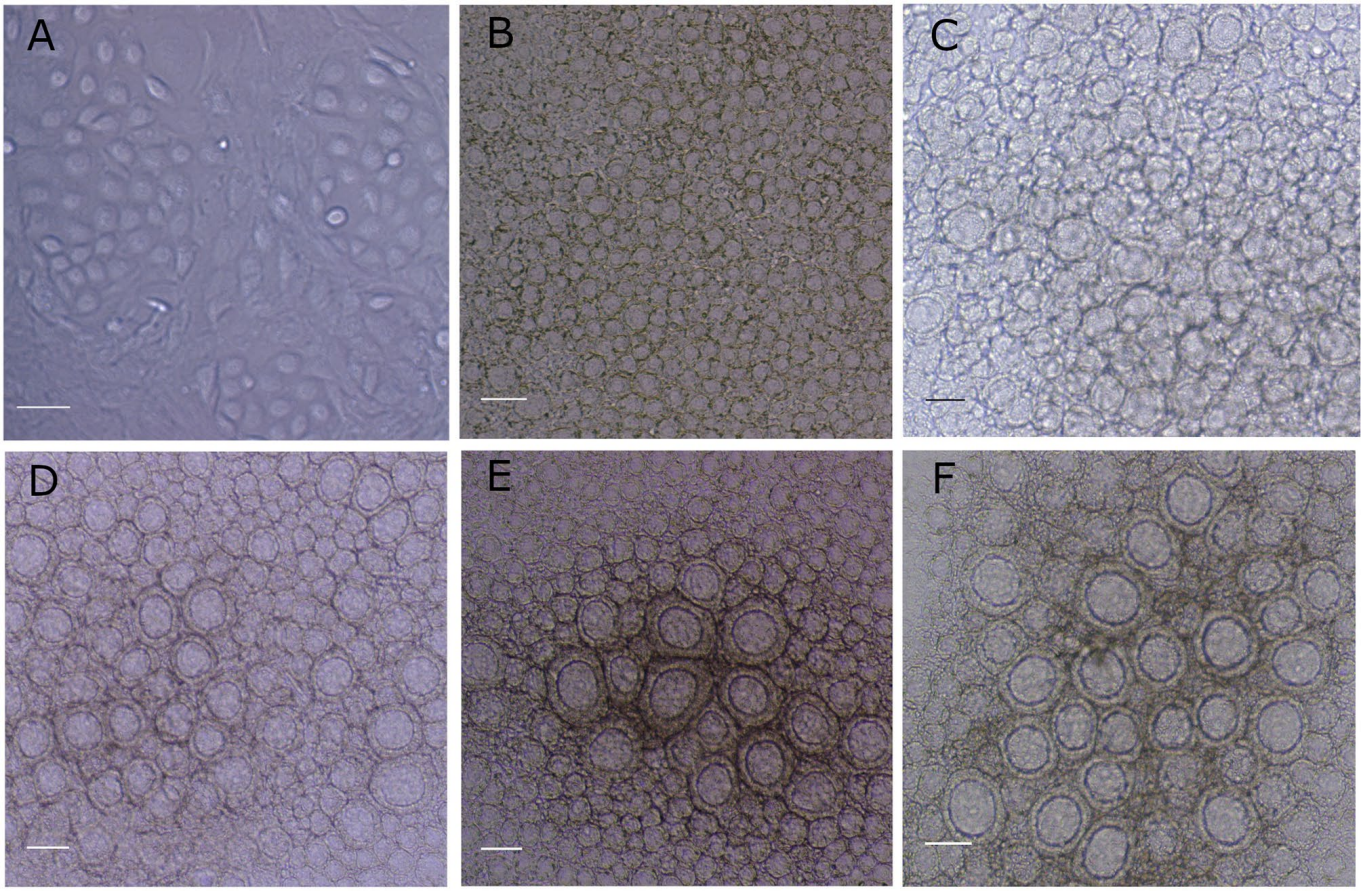
In situ microscopy contrast phase images of the progress of follicles in control conditions from ovary cultures initiated at 13.5 dpc: after 2 (**A**), 9 (**B**), 11 (**C**), 13 (**D**), 15 (**E**), and 17 (**F**) days of culture. Bars represent 50 μm.

Morohaku et al.^27^ established a protocol to obtain in vitro mouse oocytes from PGCs. However, the approach precludes a cytological^28^ and metabolic assessment of the differentiation process and analysis of the effects of potentially toxic compounds as we have performed. For these reasons, we have modified some aspects of the protocol, mainly the supports of the 3D cultures. The modifications made to the initial culture protocol did not affect the established progression of cell differentiation and folliculogenesis in mouse ovaries in the developmental window from 13.5 dpc followed by 17 days of culture (Fig. 1). The modifications of the culture procedures allowed us to perform functional analyses of the processes from 3D in vitro cultures and evaluation of the effects of potential reproductive toxicants, such as the endocrine disruptor vinclozolin (VZN), delivered in vitro during the culture process or by the culture of ovaries previously exposed in vivo*.* This approach facilitated a continuous analysis of differentiation during any window during the 17 days of culture in physiological conditions with cell–cell interactions, enabling the cytological and metabolic assessment, which are difficult to establish by other traditional methods.

To understand the follicular developmental pattern, the mouse in vitro cultures were initiated at 13.5 dpc instead of 12.5 dpc^27^ to ensure that the meiotic process had begun^29^. For the monitoring of cultures, measurements of two perpendicular diameters of each follicle in every type of exposure and control were taken every 2 days, from Day 9 to Day 17 of culture. The morphological progression of follicles from the control groups matched the progress of those analyzed in vivo^30^ (Fig. 1). Ovarian cultures from both VZN-exposed embryos in vivo (through maternal exposure) and ovaries exposed in vitro during the culture progression were assessed by measuring the diameter of follicles in whole mounted ovaries compared with the respective controls.

### In vitro exposure

**Figure 2:**
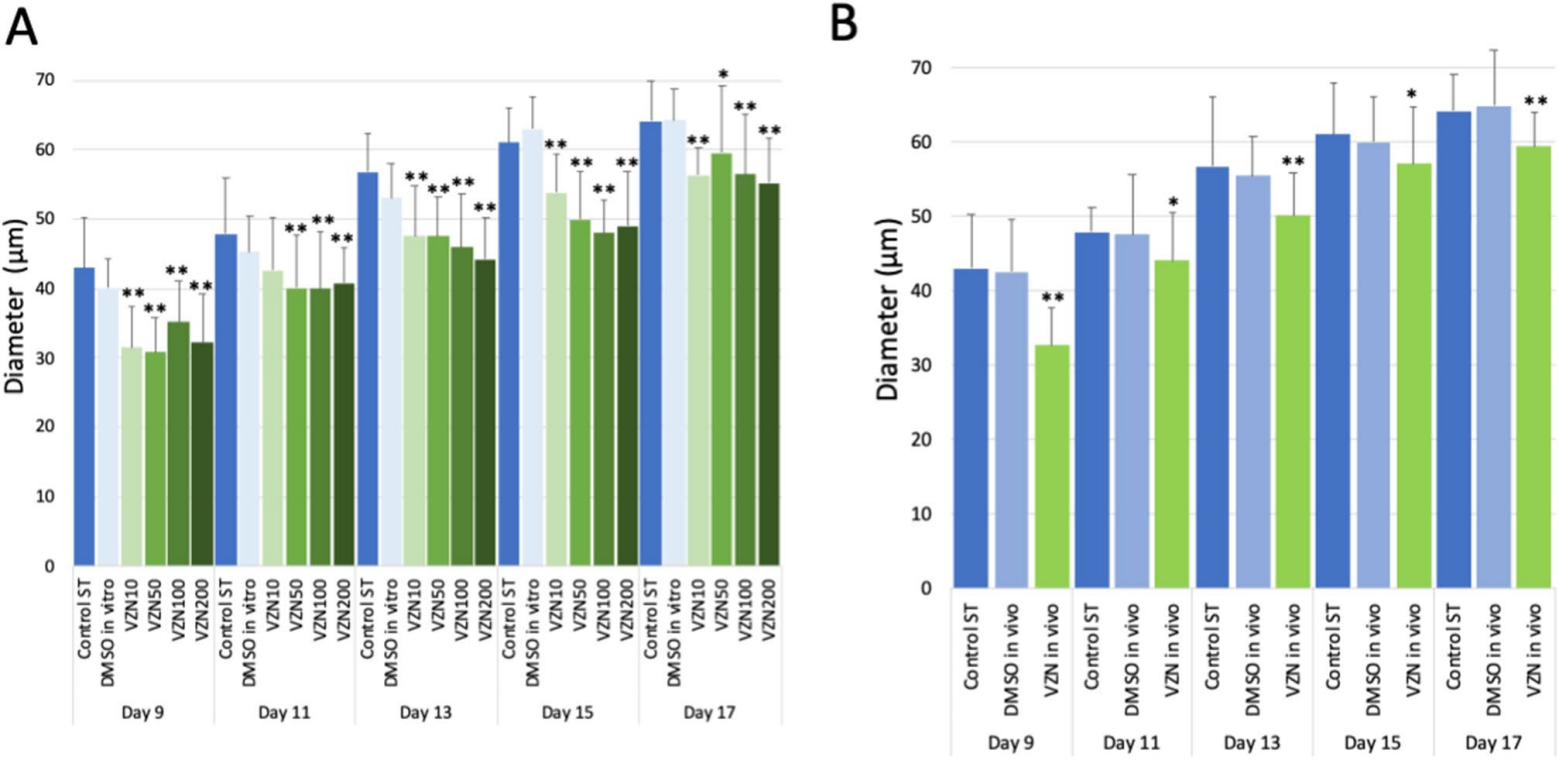
Follicle size at various days of VZN exposure. (**A**) In vitro exposure at various concentrations of VZN. Bars represent the mean of 2 to 6 replicates with each replicate containing 15–109 follicles in each experimental condition. (**B**). In vivo exposure to VZN. Bars represent the mean of 2 to 6 replicates with each replicate containing 39–152 follicles in each experimental condition. Statistical significant differences are indicated by * (*p* ≤ 0.05) or ** (*p* ≤ 0.01), in comparison to the respective DMSO controls.

First, we evaluated whether the presence of the VZN vehicle (DMSO) in the culture media alters the development of follicles. We compared the follicle size in the two types of controls: with (DMSO) and without the vehicle in the medium (control standard or ST). Our results showed that, on Day 9, the follicles growing at control ST conditions presented an average diameter of 43 μm, whereas, in the ovaries exposed to the vehicle, the average was 40.1 μm, without significant statistical difference. There continued to be no statistically significant differences between both types of controls throughout the culture progression (Fig. 2).

In order to establish a potential dose–effect correlation of VZN exposure, the analysis of follicular development was carried out with in vitro exposure to 10 μM, 50 μM, 100 μM, and 200 μM concentrations of VZN in the culture medium. Overall, we observed that follicular size evidenced a progressive and continued growth both in controls and in the follicles exposed to the various concentrations of VZN. However, follicle size was decreased at all VZN treatment levels (Fig. 2A); and, there was no correlation between dose level and follicle size during the in vitro experiment (R^2^ = 0.117). Results indicate that VZN delayed follicle progression and reduced follicle size during in vitro exposure. The lack of a dose–response during exposure suggests that the lowest dose, 10 μM, elicited the maximum attainable response.

### In vivo exposure

We evaluated the follicle diameters in ovarian explants taken from control and VZN-treated mice after 9 to 17 days in culture. No significant differences were found in the size of the follicles between the two control groups (DMSO and Control ST) (Fig. 2B). However, at day 9, follicle diameters already revealed differences between the follicles exposed in vivo to VZN compared to controls. While the controls showed an average diameter of 42.6 μm, the follicles exposed to VZN in vivo showed 32.7 μm (*p* ≤ 0.01, Fig. 2B). Size of follicles from VZN-treated mice remained significantly reduced for the duration of the culture period (*p* ≤ 0.05).These results indicate that in vivo exposure to VZN results in smaller follicles in the mouse ovary .

### Cytological analysis of germ cell progression and effect of VZN in 3D cultures

To validate the morphometric analysis of follicle progression and to evaluate potential changes in meiotic development as critical biomarkers during such progression, different functional proteins were analyzed by immunofluorescence assays.

**Figure 3:**
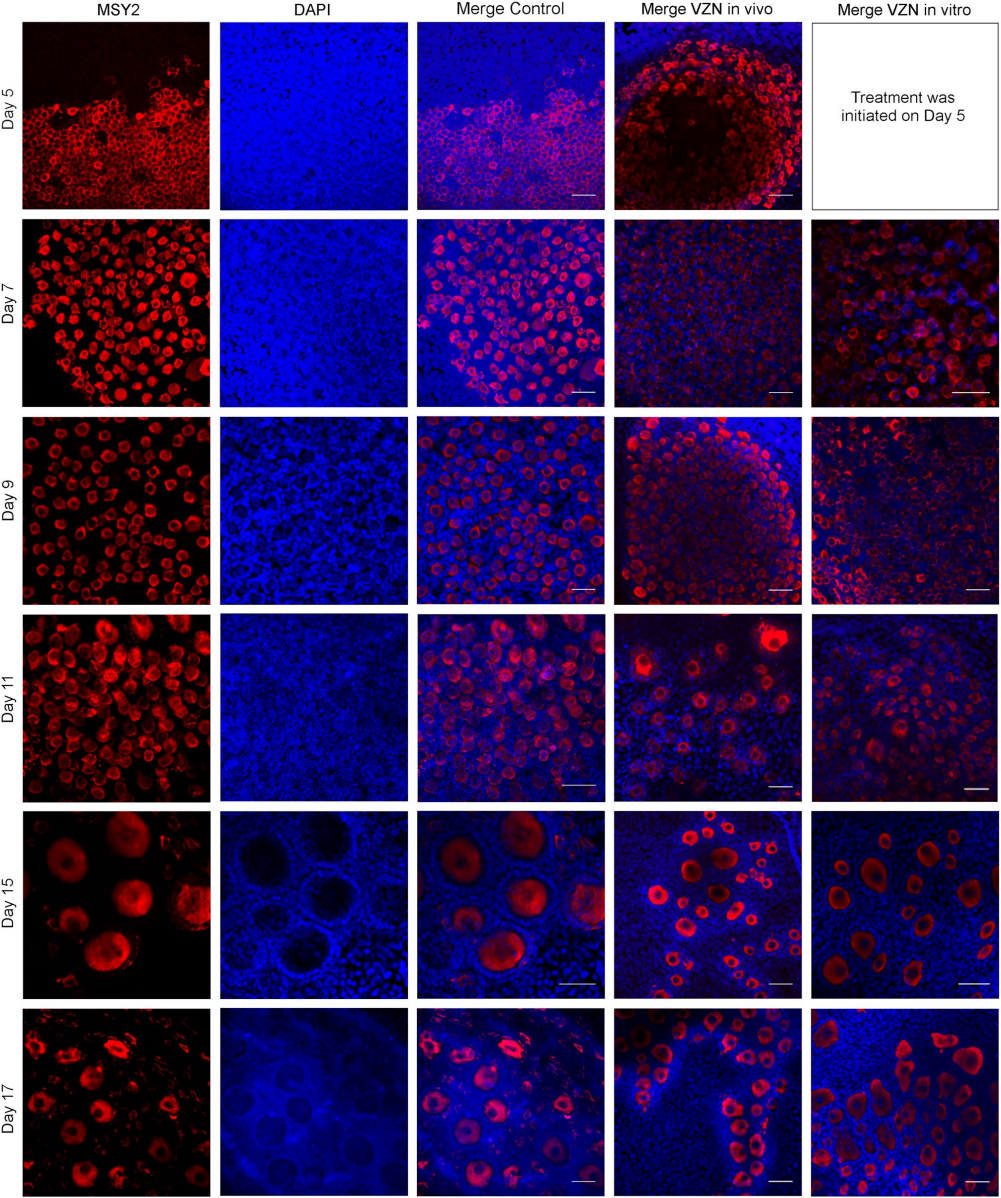
Changes in the localization of MSY2 during follicular progression during culture, DMSO controls (first three columns), in in vivo and in vitro (10 μM) exposures to VZN (as VZN exposure is initiated at day 5 of culture the first image of in vitro exposed cultures corresponds to day 7 of culture). Bars represent 50 μm.

**Figure 4:**
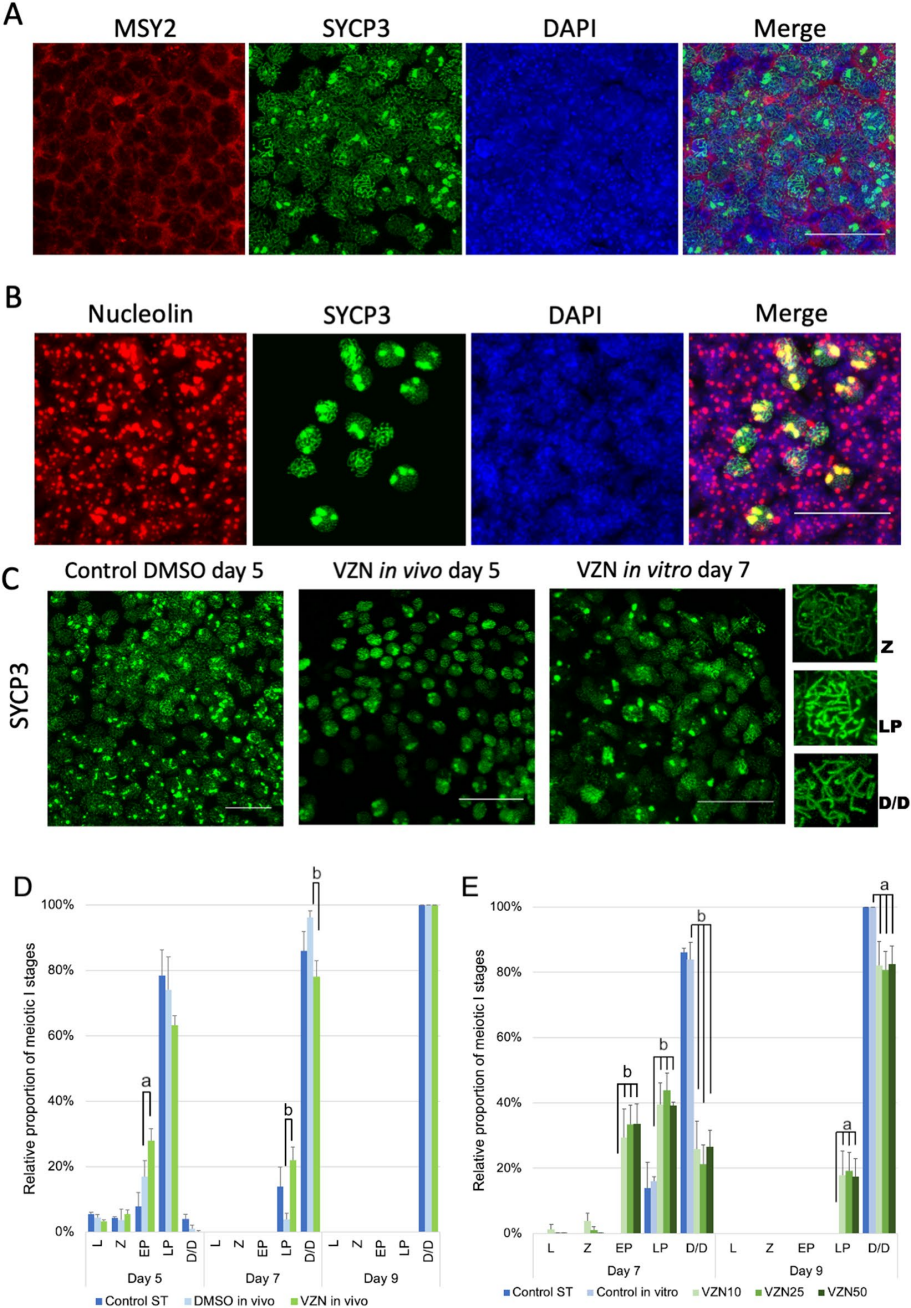
In toto immunostaining of cultured ovaries and analysis of meiotic progression. (**A**) Immunofluorescence localization of MSY2, SYCP3, and DAPI staining in meiocytes at day 5 of DMSO control culture. (**B**) Colocalization of SYCP3 and nucleolin proteins in meiotic cells SYCP3 staining. Discrete nuclear structures of lateral elements of the synaptonemal complexes are detected colocalizing with nucleoli staining during desynapsis. (**C**) Representative images of SYCP3 immunostaining of controls and VZN exposed cultures; as VZN exposure is initiated at day 5 of culture the image corresponds to day 7 of culture. Z = zygotene, LP = late pachytene and D/D = diplotene/diakinesis. Bar represent 25 μm. (**D**, **E**) Proportional distribution of different stages of meiotic cells in prophase I during the progress of ovarian cultures. (**D**) In vivo exposure to VZN*.* (**E**) In vitro exposure to VZN*.* Day 5 is not represented in this figure as the exposure to VZN in vitro began on day 5 of culture. Bars represent the mean of two biological replicates (ovaries) + SD [(**C)** 100–611 follicles per replicate; (**D**) 100–427 follicles per replicate]. Statistically significant differences respect the metabolic basal condition are indicated as: “a” correspond to *p* value ≤ 0.05 and “b” to *p* value ≤ 0.01.

We analyzed the presence of MSY2 (a germ-cell-specific member of the Y-box family of DNA-/RNA-binding proteins), initially only present in the cytoplasm of female germ cells, with a fundamental role in the regulation of mRNA stability^31^. It has been described as exclusively detectable in oogenesis after the dictyotene stage^32–34^. However, observation of our 3D cultures indicated that the presence of the MSY2 protein was already apparent from early stages of primordial follicle differentiation. In addition, a weak MSY2 signal was detected in the cuboidal granulosa cell layer (Supplementary Figure 2). This could also support the active communication between oocytes and granulosa cells^35^. Moreover, in the initial preantral stages, MSY2 localization is coherent with cystic functional organization of the oocytes and the physiologic situation of the syncytial structure in clusters, with wide cytoplasmic communication (Fig. 3, Day 5; Fig. 4A). Nevertheless, we detected no differences in the patterns of MSY2 signaling between ovaries exposed, in vitro or in vivo, to VZN and controls during culture progression, indicating that the expression of MSY2 was not altered by VZN.

Due to the differences found in the follicular size of gonads exposed to VZN in relation to controls, we decided to analyze a marker of meiotic prophase I progression of the oocytes in culture. Synaptonemal complex protein 3 (SYCP3) is an essential structural protein of the lateral elements of the synaptonemal complex (SC)^36^. By double immunolabelling with anti-SYCP3 and anti-MSY2, it was possible to cytologically validate the association between the SYCP3 marker and germinal cells (Fig. 4A).

SYCP3 is able to clearly define the cytological progress of meiotic prophase I. Surprisingly, by using SYCP3 immunodetection in toto in 3D fetal ovarian culture, we identified how SYCP3 progressively accumulated in discrete nuclear structures as the lateral elements became disorganized during desynapsis. It was reported, by temporal localization, that elements recognized by anti-SYCP3 could be localized in meiosis at nucleolar regions^37^. To verify whether the accumulated SYCP3 detected in our study was incorporated into the nucleoli, we used double immunolabeling to detect its colocalization with anti-nucleolin antibody (used as marker for the nucleolus). A high level of colocalization of both proteins was observed (Fig. 4B) (a colocalization value of 87%, quantified by Leica superposition and image comparison software). No differences in nuclear localization were detected between controls and VZN exposed ovaries. Hence, these results validated previous observations and suggested the participation of nucleoli in the pathway of disorganization of the lateral elements of SCs, at least in female meiosis.

To test whether there was a delay in the meiotic process after exposure to VZN, concomitant with the delay detected in the progression of follicle sizes in the exposed ovaries, the proportion of cells in different meiotic stages of prophase I were recorded on days 5, 7, and 9 of culture in both controls and VZN exposed ovaries. Meiotic stages were cytologically identifiable by the SC structures (Fig. 4C) in leptotene, zygotene, early pachytene, late pachytene, and late diplotene/diakinesis. The absence of structured SCs in the last stages of this differentiation was also recognized by the SYCP3 accumulation colocalized with nucleoli. VZN exposure delayed the progress of meiocytes; however, cytologically, they recovered at diakinesis after day 9 of culture. After in vitro exposure to VZN, meiotic differentiation was significantly delayed at all exposure levels (Fig. 4D,E). These analyses indicate that in vitro and in vivo exposure to VZN elicited a delay in meiotic differentiation.

### Analysis of the energy metabolism

**Figure 5:**
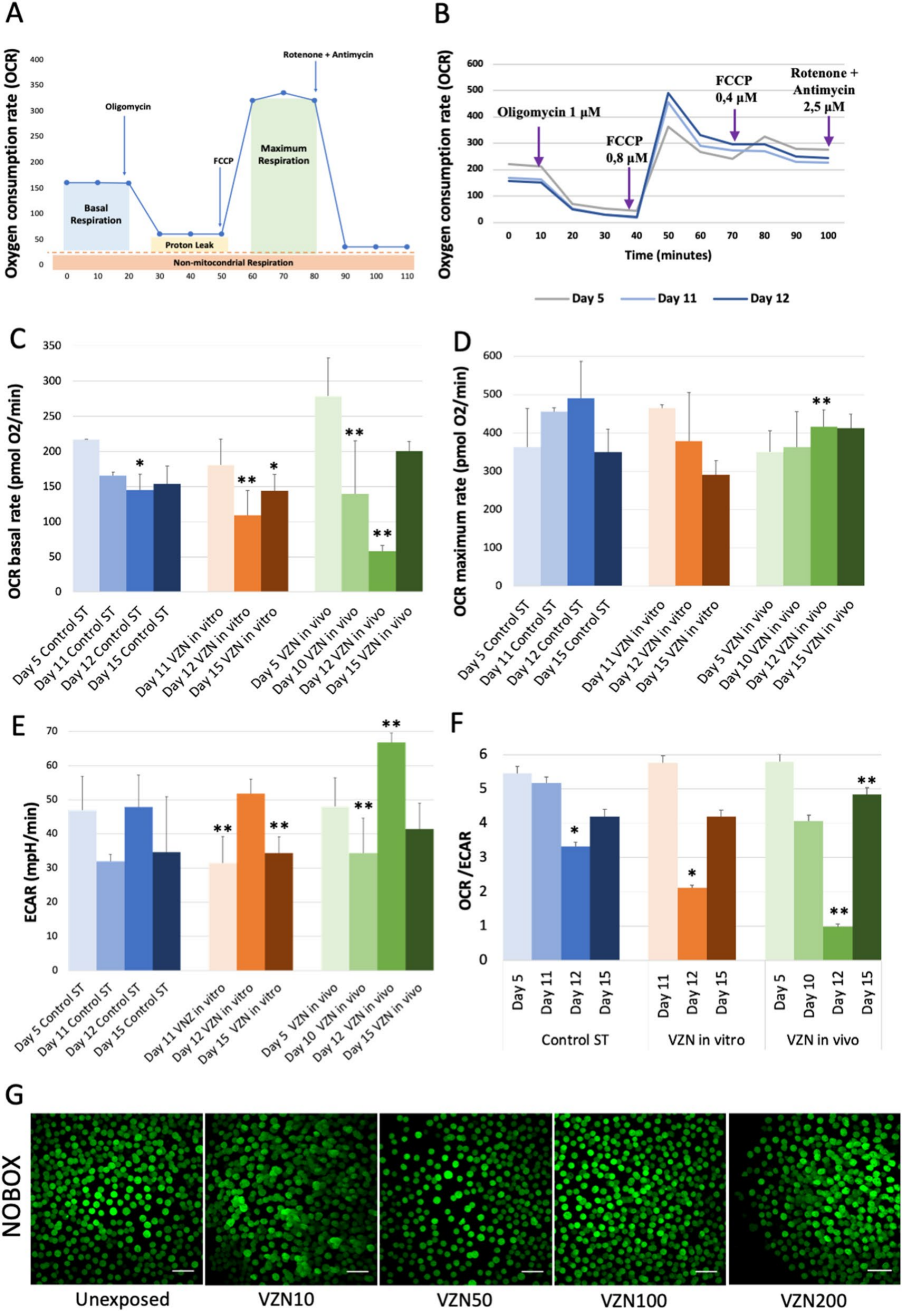
Energetic metabolism analysis. (**A**) Scheme of the parameters evaluated in the 3D cultures of fetal ovaries by the Mito Stress Test. (**B**) Mitochondrial oxygen consumption rate (OCR) on key representative days during the progress of the culture in control conditions. (**C**, **D**) Mitochondrial oxygen consumption rate (OCR) in ovaries exposed in vitro or in vivo to VZN. (**C**) Basal rate. (**D**) Maximum rate. (**E**) Quantitative measurement of basal glycolytic rate using extracellular acidification rate (ECAR) parameter, in controls cultures and in VZN-exposed ovaries. (**F**) Ratio between mitochondrial respiration and basal glycolysis in controls and in ovaries exposed in vitro or in vivo to VZN. Bars indicate the mean of 3–6 biological replicates (ovaries) ± SD. Statistically significant differences respect the metabolic basal condition are indicated as: * correspond to *p* value ≤ 0.05 and ** to *p* value ≤ 0.01 (ANOVA, Tukey’s and Dunnette’s test); (basal conditions correspond to day 5 in in vivo exposures but basal condition for in vitro exposure is day 5 control ST since it is on day 5 when experimental in vitro exposure to VZN began). (**G**) NOBOX in toto immunostaining of oocytes on day 12 of culture in DMSO controls and in vitro exposed with the different VZN concentrations experimentally used. Bar represents 50 μm.

ATP is produced in the cell by two coordinated processes, oxidative phosphorylation and glycolysis. The study of the contribution of these two processes to the cellular energy metabolism during oocyte maturation^38^ may be pivotal to understanding metabolic changes associated with proliferation, differentiation, or cellular dysfunction of female germ cells. The rates of respiration and lactate formation were measured during oocyte progression *in vitro*^39^ in exposed and control cultures (Fig. 5A, B).

Mitochondrial respiration was initially stable following in vivo or during in vitro exposure to VZN (Fig. 5C,D). On day 12 of culture, mitochondrial respiration decreased in VZN-treated gonads (Fig. 5C) that, however, did not affect the maximal respiratory capacity (Fig. 5D). Interestingly, the decrease in the basal respiratory rate was compensated by an increase in the rate of glycolysis (Fig. 5E) thus suggesting that the decrease in mitochondrial ATP production is compensated by an increase in glycolytic ATP formation. Therefore, these results reveal a metabolic change on day 12 that appears as a Warburg-like effect, that is clearly evidenced by the low OCR/ECAR ratio (Fig. 5F). To evaluate whether these metabolic changes could affect folliculogenesis during the early stages, we assessed, by immunofluorescence, the relative follicle concentration using the anti-NOBOX antibody at day 12 of culture after in vitro VZN exposure. NOBOX protein (newborn ovary homeobox gene) is crucial in early folliculogenesis^40^ and preferentially expressed in growing oocytes of the primordial follicles through metaphase II^41^. We used NOBOX as marker for growing oocytes to assess the follicle population after the different VZN exposures and controls. No apparent differences in relative follicle concentration were detected in the images (Fig. 5G). Finally, we evaluated metabolism on day 15 of culture. Interestingly and in agreement with the results by NOBOX immunofluorescence data, we observed that the OCR/ECAR ratio returns to control values, indicating that this metabolic change is transient in this period of development. Consequently, VZN exposure could metabolically affect to the oocytes in a crucial window during early differentiation associated to meiotic differentiation as we has also been detected.

## Discussion

Evaluation of endocrine disruptors and other toxicants in ovary development has been very much limited by the lack of methods for the study of in vitro processes that occur during fetal development in the whole ovary. In this study we used different methodological approaches that facilitate the evaluation of potential toxicants, which could affect early stages of female gametogenesis, by a combination of cytological and other analyses, including mitochondrial energetic metabolism.

These approaches also allow us to reveal biological processes in the context of the ovary in its entirety during development. In this context, we have determined how some components of the synaptonemal complex accumulate in the nucleoli during the meiotic progress. Some links between the synaptonemal complex and nucleoli have been previously reported^42^. Pachytene checkpoint protein 2 (Pch2) is a protein required for the checkpoint of meiotic cell-cycle progression in response to defects in recombination; it is localized into the nucleolus with suggested participation in the control of ribosomal DNA recombination. But it is not surprising that proteins that have fulfilled their function, as those that have shaped the synaptonemal complex structures, might be rapidly sequestrated in the nucleolus as other critical proteins participate in very precise and time-dependent processes, to avoid interference with other targets in the cell cycle or differentiation, generating a signaling pathway between nucleolar proteins and cellular checkpoints^43^. However this specific accumulation of SYCP3 at the diplotene stage in female meiocytes should be studied in more detail in the future.

The effects of antiandrogenic compounds, such as vinclozolin, have been widely studied during the spermatogenic process, affecting not only the individuals directly exposed but also generating transgenerational alterations transmitted by epigenetic mechanisms, including changes in DNA methylation and microRNA expressions^23,24,44,45^. However, studies on the effect of antiandrogens during development of mammalian female gonads are scarce^25,46^, particularly during the crucial process of embryonic germ cell differentiation.

In vitro culture of mouse ovaries for 12 days, starting from 13.5 dpc, as in the present study, is an equivalent period in vivo to the perinatal time, which corresponds to a key period in oocyte development, including the attrition that is initiated during PGC and meiotic prophase by apoptosis ^47–49^, but reaches the height of its expression in the perinatal period^50^.

Meiotic initiation in female germ cells is induced by high levels of retinoic acid, expressed outside the germ cells in some somatic tissues of the embryo such as in the mesonephros^51^, by stimulating the expression of *Stra8* (stimulated by retinoic acid 8)^52,53^. The meiotic progression in the performed in vitro cultured ovaries, detected by MSY2 and SYCP3, suggests that the signals necessary for such progression were not affected by the absence of the mesonephros, which we removed before culture initiation. That is, all signals involved in such commitment seem to have been transferred to the ovary previous to the start of the in vitro culture of the ovaries. However, the decline in the progression size of preantral follicles detected as an effect of VZN exposure could be interpreted as a lack of nexus between potential mesonephros signals and ovary cells. Alternatively, such deficiencies could be attributed to the metabolic pathways of the proper ovary.

The delay in meiotic progression and follicle growth seems to be affected by the antiandrogenic effect of VZN. Androgen receptors (AR) are present in the fetal ovary^54^. The meiocytes could suffer a temporary arrest in pachytene/diplotene, which is related to the smaller size detected in the primordial follicles. The role of theca cells in synthesizing androgens and transporting this signal to granulosa cells^55^, acting as the substrate for aromatase^56^, could be interfered with by the presence of antiandrogens such as VZN. In mammalian males the action of androgens is critical in spermatogenesis. Deprivation of androgens alters the first meiotic division of spermatocytes^57,58^. The meiotic checkpoint is subject to stricter controls in males than in females. The role of androgens in such a key checkpoint in oocyte meiosis is unknown^59^. Our results could indicate that the depletion of androgens by blocking AR might mediate such a difference, based on the observed effects of the antiandogenic VZN at very low concentration in vitro, able to interact with the limited number of receptors in the ovary. This is demonstrated in the various AR knockout (ARKO) mouse models, in which the phenotypes in males were more severe than in females^60^.

The role of the mitochondrial activity in oocytes is very crucial considering their increase in number during oogenesis and their stability during early development^61^. Androgen precursors may contribute to the synthesis of estrogens, mediated by aromatase (CYP19)^62^. Estrogens are involved in mitochondrial metabolism and function^63^ Mitochondria is a prevalent target of EDCs^64^ Alteration of energy metabolism by mitochondrial dysfunctions has been associated to the exposure to several EDCs^65,66^. About 800 EDCs are considered as disrupters of mitochondrial membrane potential^67^. VZN has not been previously assessed as disrupter of mitochondrial function, neither in relation with cellular alteration of folliculogenesis in early ovarian development. To assess the differentiation of the oocytes and the progress of folliculogenesis in this 3D culture model, as well as the potential effects of this EDC on mitochondria, we evaluated the energy metabolism dynamics during the differentiation process, both in controls an in VZN exposed ovaries, The results obtained in whole ovaries after 12 days of culture (equivalent to the birth developmental window) indicate a transitional change, both in vitro and in vivo*,* in the metabolic pathway with a decrease in mitochondrial respiration and increasing lactate production. However, our results demonstrated that this change was not due to any decrease in the number of mitochondria. Such anaerobic glycolysis might compensate for the reduction of oxidative phosphorylation. This change corresponds, in vivo, to perinatal age with resumption of meiosis, which is initiated in the mouse. The cumulus cells surrounding oocytes have a clear role in oocyte growth and maturation, which metabolize glucose, producing glycolytic metabolites^68^. Glucose is mainly metabolized via glycolysis in oocytes by the pentose phosphate pathway^69^. This has the consequence of relative mitochondrial inactivity to avoid oxidative stress during the differentiation process of germ cells^70^. Our results, in ovaries treated in vitro or in vivo with VZN, indicate that exposure, in early development, to this pesticide may alter the reprograming of the metabolic pathway via glycolysis and, hence, the normal differentiation in this critical period of folliculogenesis, with consequences in the ovarian reserve in adults. A similar situation of lactate increase by upregulation of the glycolytic pathway has been detected in the alteration of oocytes and follicle development in polycystic ovary syndrome patients^71^. However, the oocyte density at the time of glycolytic increase on Day 12 does not seem to be greatly affected according to our immunocytochemical experiment using the anti-NOBOX antibody and subsequent cytological analysis and could be an acute response mechanism to the toxic effect of VZN specifically at a critical point in cell differentiation.

As we show in this study, culture of the ovaries in 3D conditions from early developmental stages can facilitate a more accurate analysis of the process of oogenesis and folliculogenesis in the whole ovarian tissue environment during development.

The experimental approaches used in the analysis of the effects of toxic agents that alter germ cell differentiation and progress, might illuminate potential consequences of different toxicants in adult female fertility. The application of methodological updates introduced in the present study and the comparative results obtained in vivo and in vitro demonstrate this possibility.

## Materials and methods

### Animals

CD1 mice were the models used in this research. The experimental approaches were carried out according to the Royal-Decree Law RD53/2013 about the use and care of animals in experimentation. All assays performed had the approval of the Committee of Bioethics of Animal Experimentation of CIB-CSIC and also of the Autonomous Community of Madrid (PROEX 054/15).

### Development of female gonad cultures

The global experimental approach is schematized in Supplementary Figure 1. Embryos were obtained from pregnant females at 13.5 dpc. After dissection, the uteri of pregnant females were washed in L-15 Leibovitz media (Sigma-Aldrich) supplemented with 5% fetal bovine serum (FBS, Sigma-Aldrich) and 10 units/ml penicillin–streptomycin (Gibco). Using a stereomicroscope, we dissected the embryos and collected the gonads attached to the mesonephros, which were removed from the gonad using tungsten needles just before the seeding of the gonad. Our culture method for the ovary was based on the protocol described previously by Morahaku et al.^26,72^*.* However, in order to perform in-depth cytological, in situ immunofluorescence, and mitochondrial activity studies, we modified the method of the culture procedure depending on the process to be evaluated—follicular progression, immunofluorescence, or mitochondrial activity. The basic amendments to the original protocol were the replacement of the support of the culture from plates with Transwell-COL membranes to: 24-well polystyrene culture plates (Falcon) to evaluate the progression on follicles; 12-chamber glass slides (Ibidi) for the immunofluorescence analysis; and specific cell culture miniplates (Agilent Seahorse XFp) for assessment of mitochondrial activity of the ovaries in toto.

To evaluate the follicular progression of the culture by cytological analysis in toto, before the distribution of the gonads in the P24 plates, 125 μL of alpha-MEM (minimum essential medium) stock A (alpha-MEM, 2-O-alpha-D-glucopyranosyl-L-ascorbic acid and 0.1% vol/vol penicillin–streptomycin) was added to each well. Each gonad was divided into halves and settled in the wells for 1 h at 37 °C under 5% CO_2_, 95% air. Subsequently, wells were refilled with the same medium up to 250 μL each. After 5 days of culture (equivalent to 18.5dpc in vivo), the medium was replaced with new medium containing the estrogen receptor antagonist ICI 182,780 (R&D Systems)^27^ at a final concentration of 5 μM until Day 11 of culture (equivalent to perinatal period in vivo), which was replaced by alpha-MEM stock A until Day 17 of culture (equivalent to about 10 days postnatal). During all culture periods, the media was changed every 2 days, replacing half of the volume (125 μL) with fresh medium.

### Immunofluorescence

For immunofluorescence analysis, chamber slides with removable 12-well silicone chambers (Ibidi) were used. The day before the isolation of the gonads from the embryos, each well was covered with collagen due to the limited capacity of the explants to adhere to the glass. Collagen type I from rat tail (Merck) was diluted in 0.1 M NaHCO_3_ at a 34 μg/ml concentration, sterilized by filtration, and 200 μl of the solution was added to each well. Plates were incubated overnight at 37 °C, 5% CO_2_, 95% air, followed by replacement of collagen by supplemental alpha-MEM culture medium, washing each well two times with the same medium. The gonads, divided into halves, were settled in each well with 50 μL of alpha-MEM stock A. The explants were incubated overnight at 37 °C, 5% CO_2_, 95% air, until they adhered to the surface of the well. The medium changes were performed every 2 days, maintaining the same time period as for the ICI treatment.

### Energy metabolism

To evaluate energy metabolism, the Extracellular Flux Analyzer XFp (Agilent Seahorse) was used during the progression of the developing ovary explants. Individual ovaries (divided into three equivalent fragments) were cultivated in Seahorse XFp miniplates supplemented with alpha-MEM Stock A. The plates, containing three equidistant fragments in each well, were initially incubated in 15 μL of medium at 37 °C, 5% CO_2_, 95% air for 1 h to allow adherence of the tissue to the surface of the wells. Each well was filled with 40 μL of medium. Two of the eight wells of the plate were used as background controls for the assay. The protocol of culture was performed as indicated above.

Assessment of the bioenergetic profiles of the ovary explants was performed using the Mito Stress Test Kit (Agilent Technologies for Seahorse XFp), in which known compounds acting on the oxidative phosphorylation system were used. The mitochondrial respiration and glycolytic activity were determined from the oxygen consumption rate (OCR) and extracellular acidification rate (ECAR, a proxy for the rate of lactate production), respectively.

On the day of the analysis, the culture medium was changed to 180 μL of bicarbonate-free DMEM (Sigma-Aldrich) supplemented with glucose 5 mM, 2 mM L-glutamine, 1 mM pyruvate, 2% FBS (Sigma-Aldrich), and HEPES 5 mM pH 7.4. Cells were maintained for 1 h in a CO_2_-free incubator.

The experimental setup was based on a predesigned program called “Mito Stress Test” (Agilent) with slight modifications. After taking four measurements under basal conditions, three measurements were made in the presence of oligomycin 1 μM to inhibit mitochondrial ATP synthesis. Subsequently, measurements were made in the presence of two concentrations of the uncoupler FCCP (carbonylcyanide-p-trifluoromethoxyphenylhydrazone, 0.8 μM and 1.2 μM final) to determine the maximum respiratory rate. Finally, three measurements were made in the presence of rotenone and antimycin (2.5 μM of each inhibitor) to inhibit mitochondrial respiration.

### Exposure to vinclozolin (VZN)

To perform reprotoxicological assays, two types of exposure were carried out: a) in vitro exposures and b) in vivo exposure followed by ex vivo culture (Supplementary Figure 1). Considering that VZN can be metabolized in the liver and that in vitro biotransformation by liver microsomes has been tested at 50 μM concentration^73^, we have analyzed a wide range of VZN concentrations in ovary cultures. For in vitro exposure, cultures were treated with various concentrations of VZN (10, 25, 50, 100, and 200 μM in 0.1% DMSO) in the medium from day 5 (equivalent in vivo to 18.5) to day 11 of culture (equivalent in vivo to 19.5–20.5 dpc, that is about birth time), together with the ICI treatment. Controls were exposed to 0.1% DMSO; an additional in vitro control of 1% DMSO was also evaluated. In vitro doses of VZN were based on previous analyses testing steroidogenesis in exposed culture cells at non-toxic concentrations ranged from 0.3 to 300 μM VZN in the same vehicle (0.1% DMSO)^22,74^.

In rodents, the developmental lowest observed adverse effect level (LOAEL) was established at 50 mg/kg/day^75^. In the present study, to evaluate in vivo exposure, pregnant female mice were exposed, by drinking water, to the LOAEL dose of 50 mg/kg bw per day of VZN (Sigma) dissolved in DMSO (Sigma) (1.1 g/kg/day) from plug detection (0.5 dpc) until euthanasia at 13.5 dpc. The same dosage of DMSO was used as a control (as well as controls without DMSO [control ST]). This VZN dosage has been previously used in female mice to investigate the effects during embryogenesis^76^, although effects in rodents has been detected at 100–200 mg/kg bw/day of VZN^77–79^, an increase in polycystic ovaries in F3 offspring generation after 100 mg/kg bw/day exposure in F0 was also detected^80^. Cultures were performed as indicated above.

### Cytological and morphometric analysis of in vitro follicular progression

Follicle growth is good biomarker of follicle development^81^. During follicular development, photographs were taken using in situ microscopy phase contrast along the culture progression, starting in all cases from 13.5 dpc ovaries, with a Nikon Eclipse TE300 microscope equipped with a Nikon DXM1200 camera and the ACT-1 for DXM1200 and DXM1200F programs. Follicle size was analyzed by measuring and averaging two perpendicular diameters of all follicles displayed in randomly selected microscopic areas of the cultures^31^, using the ROI Manager tool from the ImageJ program. A total of 2775 follicles were measured. An average of 69 follicles were analyzed per each selected day after culture initiation and exposure. Dispersion analysis was carried out to confirm that the number of follicles analyzed did not show correlation with data dispersion. That is, samples showed similar dispersion although the number of cells analyzed was different.

### Immunofluorescence assay

The explants cultured directly on slides with removable 12-well silicone chambers as indicated above, were rinsed in phosphate-buffered saline (PBS) with 0.1% Triton-X 100 after removing the culture medium and the silicone wells. Fixation of the tissues was performed by sequential incubation in 1%–2%–4% paraformaldehyde in PBS with 0.1% Triton X-100 for 1 h at room temperature. Then, the tissue was rinsed three times with PBS/0.1% Triton-X 100, followed by blocking with 5% bovine serum albumin during 30 min, incubated overnight with primary antibodies at 4 °C in blocking solution in a humidified chamber, washed three times for 15 min, and incubated 1 h with secondary antibodies. Finally, the silicone chambers were removed, and the slides were washed 3 times for 15 min and mounted in DAPI UltraCruz mounting medium (Santa Cruz Biotechnology). The primary antibodies were: mouse anti-SYCP3 (Santa Cruz Biotech.) at 1:500 dilution in PBS; rabbit anti-MSY2 (gift from R. Shultz, Univ. Pennsylvania) at 1:1000; goat anti-NOBOX (gift from A. Rajkovic, Univ. of Pittsburgh) at dilution 1:100; and rabbit anti-nucleolin (Abcam, 1:100). Secondary antibodies were: Alexa 488 goat anti-mouse (Jackson ImmunoResearch) 1:700; Alexa 568 goat anti-rabbit (Molecular Probes) 1:700; and Alexa 488 rabitt anti-goat (Molecular Probes) 1:700.

Images from whole mounted preparations of the ovaries were collected using fluorescence microscopy (Leica) and confocal laser (Leica SP5). Colocalization of the proteins marked with the anti-SCYP3 and anti-nucleolin antibodies was performed with the Leica Application Suite of image superposition and comparison software (LasX).

### Meiosis progression

To study the meiotic progression in prophase I oocytes, we analyzed the different stages of the cells during Days 5, 7, and 9 of culture, in the case of in vivo exposure to VZN, and Days 7 and 9 in the case *of *in vitro exposure (as the in vitro exposure began on Day 5). The assays were carried out by counting meiocytes at different stages, immunolabeled with anti-SYCP3 antibody in whole mounted preparations of cultured ovaries. An average of 368 meiocytes were studied per day and exposure; a minimum of 100 follicles were analyzed under each condition.

### Statistical analysis

In the cytological and morphometric assay, data was analyzed by the Kruskal–Wallis and bilinear regression, used to determine the dosage of VZN. To compare the variables among groups exposed to VZN and controls in the meiosis progression assay, the statistical analyses were carried out by one-way analysis of variance (ANOVA) with Bonferroni post hoc analysis. In the energetic metabolism analysis, one-way ANOVA test was used followed by Tukey´s and Dunnette post hoc analysis. The data was processed using Seahorse Wave Desktop Software. SPSS Statistics software was used for all statistical analyses.

## Supplementary information

Supplementary Legends.

Supplementary Figure 1.

Supplementary Figure 2.
