# Supplementary material for: The antiandrogenic vinclozolin induces differentiation delay of germ cells and changes in energy metabolism in 3D cultures of fetal ovaries
Source: Sci Rep. 2020 Oct 22;10:18036. doi: 10.1038/s41598-020-75116-3 (PMC7582921; doi:10.1038/s41598-020-75116-3)
Supplement: Supplementary file 1 — Supplementary Legends. [file 41598_2020_75116_MOESM1_ESM.docx]

**Supplementary information**

**The antiandrogenic vinclozolin induces differentiation delay of germ cells and**

**changes in energy metabolism in 3D cultures of fetal ovaries**

Silvia González-Sanz^1^, Odei Barreñada^1^, Eduardo Rial^2^, Miguel A. Brieño-Enriquez^3#^,

and Jesús del Mazo^1#*^

^1^Department of Cellular & Molecular Biology. Centro de Investigaciones Biológicas

Margarita Salas (CIB-CSIC) Ramiro de Maeztu, 9. 28040 Madrid. Spain.

^2^Department of Structural and Chemical Biology. Centro de Investigaciones Biológicas

Margarita Salas (CIB-CSIC) Ramiro de Maeztu, 9. 28040 Madrid. Spain.

^3^Magee-Womens Research Institute, Department of Obstetrics, Gynecology and

Reproductive Sciences. University of Pittsburgh School of Medicine, USA.

^#^These authors contributed equally to this study.

^*^ Corresponding author: jdelmazo@cib.csic.es

**Supplementary figures**

**Supplementary Figure 1**: Schematic experimental approaches. Green arrows indicate processes in which the ovaries were not exposed to VZN and red arrows those in which the ovaries were exposed to VZN (*in vitro* or *in vivo*). Embryo images of 13.5 dpc were recorded before gonad dissection.

**Supplementary Figure 2:** Localization of MSY2 in the cytosol of oocytes and in surrounding cuboidal granulosa cells including DAPI staining of the overall ovarian morphology. Bar represents 25 μm.
