# Supplementary figures and images for: The antiandrogenic vinclozolin induces differentiation delay of germ cells and changes in energy metabolism in 3D cultures of fetal ovaries
Source: Sci Rep. 2020 Oct 22;10:18036. doi: 10.1038/s41598-020-75116-3 (PMC7582921; doi:10.1038/s41598-020-75116-3)

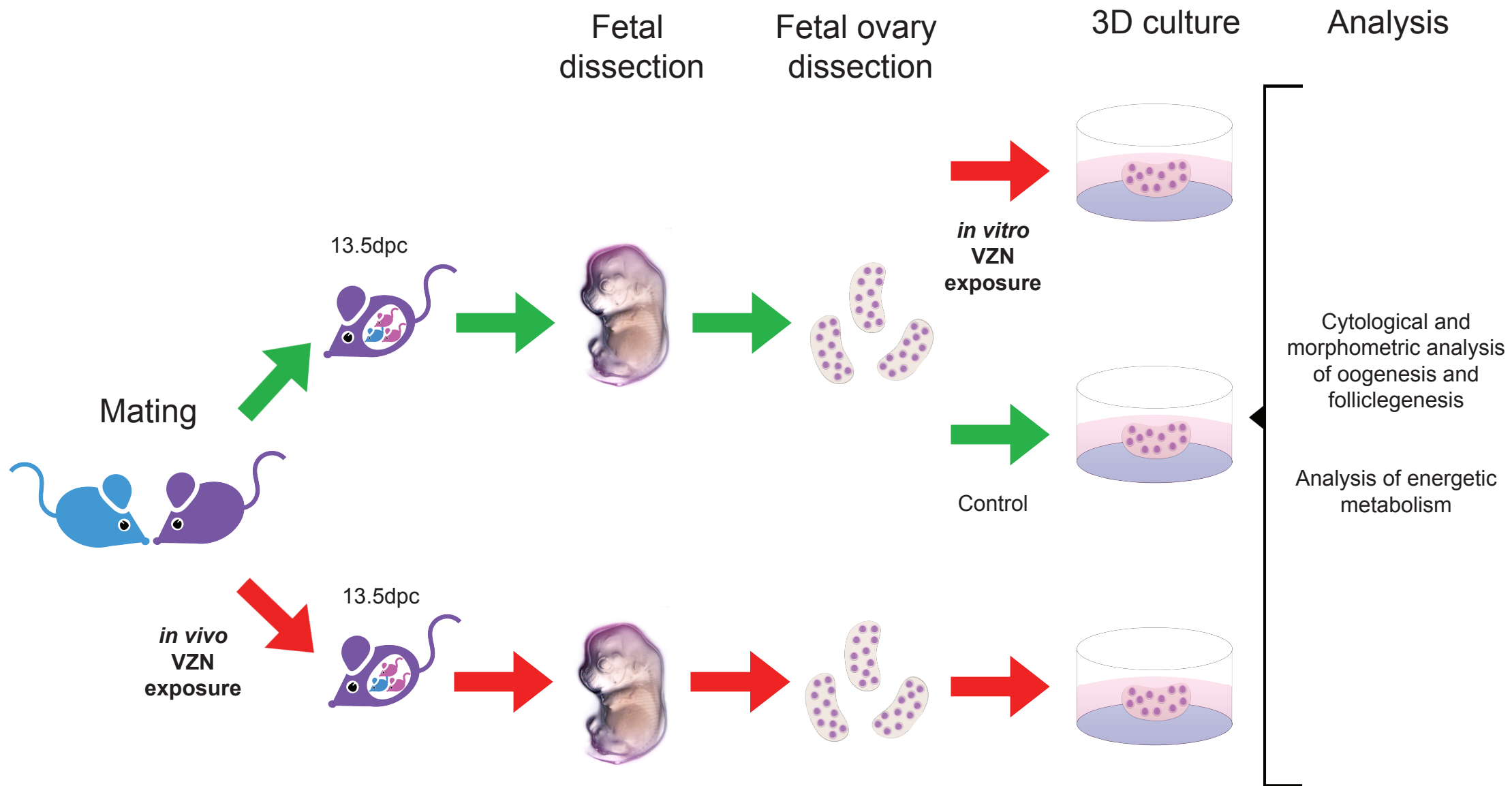

Supplement: Supplementary file 2 — Supplementary Figure 1. [file 41598_2020_75116_MOESM2_ESM.pdf]

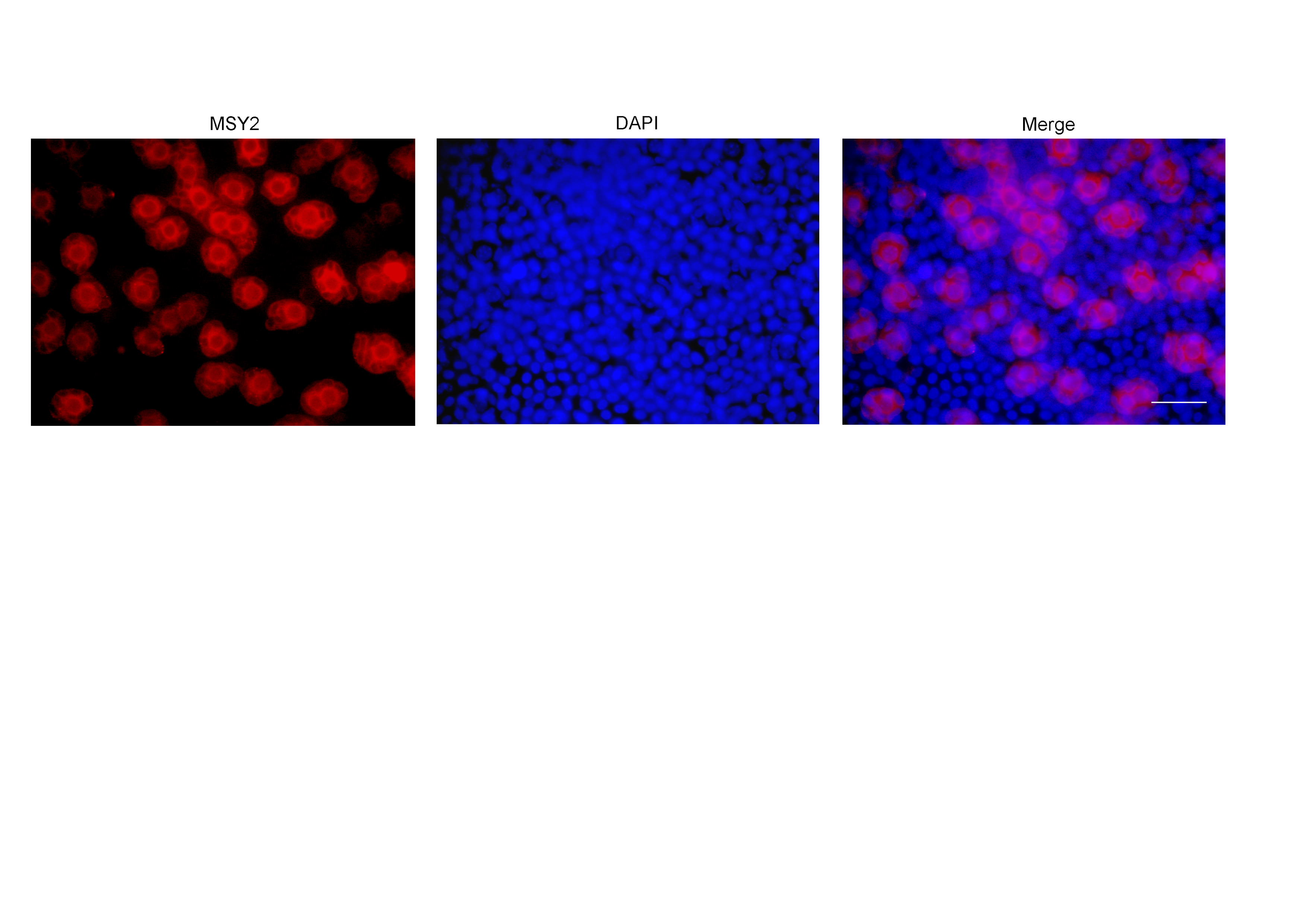

Supplement: Supplementary file 3 — Supplementary Figure 2. [file 41598_2020_75116_MOESM3_ESM.tiff]
